# Environmental and Genetic Effects on Pigment-Based vs. Structural Component of Yellow Feather Colouration

**DOI:** 10.1371/journal.pone.0036640

**Published:** 2012-05-10

**Authors:** Jana Matrková, Vladimír Remeš

**Affiliations:** Department of Zoology and Laboratory of Ornithology, Palacký University, Olomouc, Czech Republic; Arizona State University, United States of America

## Abstract

**Background:**

Carotenoid plumage is of widespread use in bird communication. Carotenoid-based feather colouration has recently been shown to be dependent on both pigment concentration and feather structure. If these two components are determined differently, one plumage patch may potentially convey different aspects of individual quality.

**Methodology/Principal Findings:**

We evaluated the effects of genetic and environmental factors on carotenoid-based yellow breast colouration of Great Tit (*Parus major*) nestlings. By partial cross-fostering, we separated the genetic and pre-natal vs. post-natal parental effects on both the structural and the pigment-based component of carotenoid-based plumage colouration. We also simultaneously manipulated the post-hatching environment by brood size manipulation. The structural component of nestling colouration reflected features of female colouration. On the other hand, the pigment-based component was more affected by rearing conditions presumably representing food quality. While the structural component was related to both origin- and environment-related factors, the pigment-based component seemed to be environment-dependent only. These results support the notion that pigment-based and structural components of feather colouration are determined differently.

**Conclusions/Significance:**

Chromatic and achromatic components of carotenoid-based feather colouration reflected different aspects of individual quality and history, and thus may potentially form a multicomponent signal.

## Introduction

Signal design is a very important component of animal communication. In visual signals, colouration and overall patterning may be critical for signal efficiency [1]. Thus, factors affecting individual components of a visual signal are of high importance for our understanding of signal function and evolution. In birds, plumage colouration is a conspicuous and versatile trait with important signalling functions [2]. Both feather structure and pigments can determine plumage colouration and are thus important drivers of signal design and efficiency [3]. Structural colours are generated by physical interaction of light waves with tissue matrices, while pigment-based colours are determined by molecular structure of the pigment and its density [4]. Interactions between light-scattering tissue structures and pigment molecules are common in animal colouration, but only one component is typically considered at a time [5]. Accordingly, in behavioural and evolutionary studies, structural and pigment-based plumage colours have usually been treated as distinct. For example, carotenoid-based colouration has been considered to be fully pigment-based (e.g. [6]–[8]).

Carotenoids are frequently deposited into plumage causing yellow to red hues [9].

However the colour properties of carotenoid-based plumage do not depend solely on the carotenoid content. Recent research has revealed that carotenoid-based feather colouration is produced by the interaction of pigment and feather structure [3], [10]. The keratin feather structure uniformly reflects light across all wavelengths, creating the white background. The yellow to red chroma is produced by carotenoids that absorb light in a specific area around 400–500 nm thus eroding reflectance in this wavelength band. Carotenoids cannot produce yellow colour without the white reflective background, because they do not significantly reflect light themselves. A combination of the reflectance of the white feather structure across all wavelengths and the absorbance of specific wavelengths by carotenoid pigments is therefore necessary to produce the yellow to red chroma [3], [10].

Experimental studies have firmly established plasticity of carotenoid-based feather colours in relation to a variety of environmental factors. They reflect the nutritional state of a bird, its access to dietary carotenoids [11]–[13], parasite load and the activation of the immune response [8], [13] Carotenoid supplementation has shown that variation in carotenoid content in feathers affects colour properties of these signals [10], [14], [15]. Similarly to carotenoid-based colouration, colours based on feather structure can be affected by hormones, parasites, nutritional conditions during moult and the moult speed ([16]–[18], [8], [19]–[21]; but see [22]). Thus a critical question arises as to what part of the carotenoid signal plasticity is due to the variation in pigment content and what part is due to the variation in feather structure. This leads to the further query as to which factors drive variation in these two components. These questions have been rarely investigated [10], [23], [15], [24].

Here we studied carotenoid-based feather colours of nestlings of the Great Tit *Parus major*, which have become an important model system for the investigation of signal function and plasticity. A wide spectrum of environmental and genetic determinants of carotenoid colouration have been evaluated in both Great Tit [25]–[34], [14], [35]–[38] and the Blue Tit *Cyanistes caeruleus* [39]–[42], [31], [43]–[48], [33], [21], [49], [50] offspring. However, only a few studies discriminated between the structural and the pigment-based component of carotenoid colouration in titmice [23], [51], [52], [15], [24], or in any other species of bird [10]. Consequently, we lack a firm understanding of the relative plasticity of these two components of feather colouration. To our knowledge, no study to date has assessed the genetic determination of these two components separately in juvenal plumage.

Thus, here we evaluated effects of genetic and environmental factors on both the structural and the pigment-based component of yellow carotenoid-based breast colouration of Great Tit nestlings. By partial cross-fostering, we separated the genetic and pre-natal vs. post-natal parental effects on nestling plumage colouration. We also simultaneously manipulated the post-hatching environment by brood size manipulation to expose nestlings to rearing conditions of different quality. Additionally, we directly measured a number of environmental factors (hatching date, egg yolk antioxidants, feeding rate of parents), as well as carotenoid-based breast colouration of females. We analysed possible effects of these factors separately on pigment-based and structural components of nestling carotenoid-based colouration. We show that the structural and the pigment-based component of carotenoid-based colouration reflect different aspects of the rearing environment and the genetic background of nestlings.

## Methods

### Ethics Statement

Standard methods in capturing and handling birds used in the research of cavity-nesting passerines were used. Adults were captured in the nest-box. They were handled for as short time as possible to minimise any distress. The smallest number of feathers possible to obtain reliable results were plucked, which was based on a previous methodological study [53]. This study complies with the current law of the Czech Republic. We had all necessary permits for this study, and it was approved by the Ethical Committee of Palacký University.

The study was permitted by: Project of experiment according to Section 12, the Decree No. 311/1997 Coll., on the breeding and use of experimental animals (Faculty of Science, Palacký University Olomouc, ID 45979/2001-1020), ringing licence (Vladimír Remeš, ID 1051), the decision on the derogation according to Section 5b (Conditions for Derogations in Protection of Birds), the Act no. 114/1992 Coll., on Nature and landscape protection (Without ID, approved by The Department of Environment, Municipality of Olomouc).

### Study Site

This work was conducted on three adjacent nest-box plots (188 nest-boxes in total, their design is described in [54] – study site Olomouc) in a deciduous forest near Grygov (49°31′N, 17°19′E) in the eastern Czech Republic. The forest is dominated by lime *Tilia* spp. and oak *Quercus* spp. with interspersed ash *Fraxinus excelsior*, common alder *Alnus glutinosa* and common hornbeam *Carpinus betulus*. Nest-boxes were placed about 1.5 m above ground. These nest-boxes were, besides Great Tits, inhabited by Blue Tits, Collared Flycatchers *Ficedula albicollis*, and Nuthatches *Sitta europaea.*

### General Fieldwork

Field work was carried out in 2005 from early April until mid-June when the nesting of Great Tits terminated. We checked the nest-boxes every other day to record laying of the first egg. Subsequently, we numbered the eggs with a water proof felt pen. Before birds started the incubation we removed one egg from each clutch. The order of the removed egg in laying sequence was on average 4.2 (ranged 3 to 5). The removed eggs were weighed and stored in –20°C for subsequent analyses.

One day after the clutch was completed we weighed the whole clutch on a digital balance to the nearest 0.01 g. At the end of incubation, we visited the nests daily to find out the day of hatching. The day when the first nestling hatched was considered the day 0 of the brood age. When the young were six days old, we ringed them with an aluminium ring. On day 14, we measured the right tarsus with a digital calipper to the nearest 0.01 mm, the right wing (the longest primary) with a ruler to the nearest 0.5 mm, and weighed the young on a digital balance to the nearest 0.1 g. For each bird, we took from 10 to 15 yellow feathers from the upper right part of the breast for later spectrophotometric analysis.

We captured females in 43 out of 46 nest-boxes during the nestling period (the median age of the young  =  7 days). Due to time constraints, we were not able to capture males. We measured the tarsus and wing length of the females, weighed them, and removed breast feathers for the analysis in the same way as in the young. We determined the age of the birds based on their plumage as one year old or older [55].

When nestlings were eight days old (except 5 nests; range 6 to10 days), we placed a video camera about five meters in front of each nest-box on the ground. Parental activity was recorded for 90 minutes in the morning hours (from 7∶30 to noon). We discarded the first 15 min of recording and quantified the feeding rate per hour.

### Analyses of Yolk Antioxidants and the Feather Colouration

We analysed the concentration of lutein, zeaxanthin, vitamin E and vitamin A in the egg yolks as in [56]. Briefly, we extracted the samples by acetone/methanol method and injected them into the HPLC system. We used an Agilent 1100 Series HPLC system (Agilent Technologies, Waldbronn, Germany). LC separation was carried out on a Zorbax SB-CN rapid resolution (75×4.6 mm, particle size 3.5 µm), reversed-phase column (Agilent Technologies, USA). The mobile phase consisted of 0.05 mM (v/v) ammonium formate and methanol. The flow rate was 0.7 mL/min and typically 10 µL aliquots were injected into the column. The column oven temperature was set at 30°C.

According to the standard procedure [57], we quantified the reflectance spectra of yellow feathers sampled from the breast. We used on average 10 feathers from each bird (10 ± 3.1 feathers; mean ± SE) We used Avantes AvaSpec-2048 fiber optic spectrometer together with AvaLight-XE xenon pulsed light source and WS-2 white reference tile. The probe was used both to provide light and to sample the reflected light stream and was held perpendicular to the feather surface. Feathers were arranged on a black, nonreflective surface so that they overlapped extensively. We took three and five readings per a young and a female, respectively, each from a different part of each set of feathers. We obtained reflectance (in %) from the wavelength of 320 to 700 nm in 1-nm increments.

We wanted to test the effect of the origin and rearing environment on both the pigment-based and the structural components of the feather colouration of Great Tit young. To assess the structural component of the feather colouration, we calculated ***background reflectance*** of the feathers as the sum of absolute reflectance between 575 and 700 nm [15]. In this area, the light reflected by the feather structure is unaffected by the carotenoid content, because carotenoids absorb light at lower wavelengths [15], [57]. Accordingly, experimental carotenoid extraction from American Godfinch feathers only weakly affected the reflectance above 575 nm [3], [10]. The ***absolute carotenoid chroma*** (–1×(R_400−515_/R_575−700_)) represented the pigment-based component of the colouration. Absolute carotenoid chroma correlates with the carotenoid content of feathers [15]. For convenience, we made the values of absolute carotenoid chroma negative (see the multiplication by –1 above) so that the correlation with feather carotenoid content was positive in sign. Accordingly, our absolute carotenoid chroma was strongly positively correlated with the carotenoid chroma (R_700_– R_450_)/R_700_) of the feathers in both the young (r = 0.97, P<0.001, n = 373) and females (r = 0.99, P<0.001, n = 43). At the same time, carotenoid chroma was shown by theoretical modelling to directly reflect the amount of carotenoids in feathers [57] and correlated positively with feather carotenoids in the Great Tit in previous studies [14], [35]. These correlations suggest that our absolute carotenoid chroma was a good indicator of the carotenoid content of yellow breast feathers. Absolute carotenoid chroma correlated only weakly with background reflectance in nestlings (r = 0.21, P<0.001, n = 353) and not at all in females (r = –0.06, P = 0.71, n = 43).

In statistical analyses, we used the average values of colour characteristics calculated from the five readings from each set of feathers for females and from the three readings for nestlings.

### Cross-fostering and Brood Size Manipulation

One day after the first young in the clutch hatched, we performed a partial cross-fostering experiment with simultaneous brood size manipulation. Cross-fostering was performed between pairs of nests – dyads. We assigned nest to dyads based on their same hatching day and when possible also their same clutch size. There was no difference in clutch size in 13 dyads, in 8 dyads nests differed by one egg, and in 1 dyad by two eggs.

We weighed all young on a digital balance to the nearest 0.01 g. Within the nest of origin, we ranked them according to their weight from the heaviest to the lightest. Beginning either from the first or the second heaviest nestling of each nest, we swapped every other young between the two nests of the dyad. The rest of the young stayed in their nests of origin. In this way, we exchanged either even- or odd-ranked nestlings (according to the mass hierarchy) within the dyad. The choice of odd- or even-ranked nestlings to be exchanged alternated between subsequent dyads. The mass hierarchy of nestlings after cross-fostering was kept close to the original mass hierarchy before cross-fostering. The weight of the cross-fostered and non-cross-fostered young on the day of cross-fostering did not differ (LMM, nest of origin as a random factor: F_1,336_<0.01, P = 0.94).

We intended to manipulate brood size by two young. Thus, during the cross-fostering, in one nest of each dyad we randomly chose one extra nestling and took it also to the foster nest. In the second nest of the dyad, we additionally randomly chose one nestling not to be cross-fostered and left it in its nest of origin. In this way we increased the brood in the second nest of the dyad by two nestlings, leaving the first nest two nestlings short. The design of cross-fostering and brood size manipulation is available in supplementary material (see fig S1). Brood size manipulation significantly affected brood size on the day of feather sampling (t test: t_40_ = 7.67, p<0.001, R^2^ = 0.60, enlarged brood: 10.7±0.31 young; reduced brood 7.3±0.31 young; mean ± SE) and nestling size. Nestlings from enlarged broods were on this day lighter than nestlings from reduced broods (16.21±0.169 g vs. 17.04±0.174 g; LMM, nest of origin and nest of rearing as random factors: F_1,16.8_ = 13.88, P = 0.002), they had shorter tarsi (22.66±0.073 mm vs. 22.87±0.078 mm; F_1,16.0_ = 6.21, P = 0.024) and were in worse condition (–0.24±0.134 vs. 0.39±0.140; F_1,8.7_ = 18.7, P = 0.002). On average the whole process of cross-fostering took 13.4 minutes per nest-box (range 9 to 20 minutes). At the beginning of the cross-fostering experiment, we marked the nestlings by clipping down feathers on the head and back for their further individual recognition. In cases with unequal brood size within the dyad, the procedure was adjusted so that the result was the same as in dyads with the same number of the young (i.e., mass hierarchy was kept similar to original nests and brood size was manipulated by two young). Larger broods become enlarged as often as smaller broods in dyads with unequal brood size (4 and 5 cases, respectively).

### Statistical Analyses

To identify effects of individual factors on the pigment-based and the structural component of the nestling feather colour, we fitted linear mixed models (LMM) with *background reflectance* and *absolute carotenoid chroma* as dependent variables. *Nest of origin* and *Nest of rearing*, both nested within the *Dyad*, and *Dyad* itself, were entered as random factors. *Dyad* represented a matched pair of cross-fostered nests and accounted for the variability due to the pair of nests. *Nest of origin* accounted for any pre-cross-fostering effects and included genetic effects, prenatal maternal effects and environmental effects early after hatching up to one day of age when cross-fostering took place. *Nest of rearing* accounted for post-cross-fostering effects, i.e. the rearing environment including the parental care.

As fixed factors, our models included *background reflectance* and *absolute carotenoid chroma* of genetic and foster mothers, their *age*, total *feeding rate per capita* (summed feeding rate of both parents per hour per nestling at day 8), *yolk antioxidants*, and *brood size manipulation* (categorical variable, level + or –). Brood size itself was not included in the model, as it tightly correlated with both *nestling condition* (r = –0.49, P<0.001, n = 42) and feeding rate per capita (r = 0.70, P<0.001, n = 43). We wanted to avoid collinearity of our predictors and thus we modelled possible effect of the brood size by including these two factors. Yolk antioxidants were represented by the first principal component (PC1) from a principal component analysis (PCA). PCA was run on concentrations (µg g^–1^) of vitamin A, vitamin E, lutein and zeaxanthin in egg yolk, and PC1 explained 69.1% of the total variance (factor loadings: vitamin A: 0.77; vitamin E: 0.76; lutein: 0.88; zeaxanthin: 0.89; all factors log_10_-transformed). The results for models with PC1 did not differ from models where these antioxidants were included separately (results not shown). The model further included *nestling condition* (residuals from the regression of body mass in g on tarsus length in mm at day 14) and *hatching date*. To control for feather development in nestlings, we included the *feather length* (average length of five breast feathers) as a fixed factor. Background reflectance significantly correlated with feather length (r = 0.15, P = 0.008, n = 296), whereas absolute carotenoid chroma did not (r = –0,10, P = 0.083, n = 296).

Nestlings in four nests did not survive until the feather sampling and final measurements. For the delayed feather development, we excluded from the analyses extremely small (n = 4) nestlings and nestlings hatched after cross-fostering (n = 25). Further nestlings were excluded from the analyses due to missing data (e.g. female was not captured, unsuccessful antioxidant analysis etc.). As a result, 296 nestlings in 40 nests and 21 dyads were included into analyses.

We tested LMM with procedure MIXED in SAS. The covariance parameters were estimated by the REML covariance method. We used the COVTEST statement to produce asymptotic standard errors and Wald Z-tests for the covariance parameter estimates. Variables were checked for normal distribution. Residuals from each model were checked to conform to the requirements of normal distribution, equal variance, and linearity [58]. To compare the size of effects both within and between models, we used standardized regression coefficients. We standardized all continuous input variables by subtracting mean and subsequently dividing by two standard deviations. Due to this standardization, effect sizes for continuous and categorical factors are directly comparable [59]. Finally, to compare mean effect size of specific subsets of factors regardless of the direction of individual effects, we summed absolute values of their standardized regression coefficients. All statistical analyses were conducted in SAS 9.2 (LMM) and JMP 7.0.1 (other tests and data transformations).

## Results

**Figure 1:**
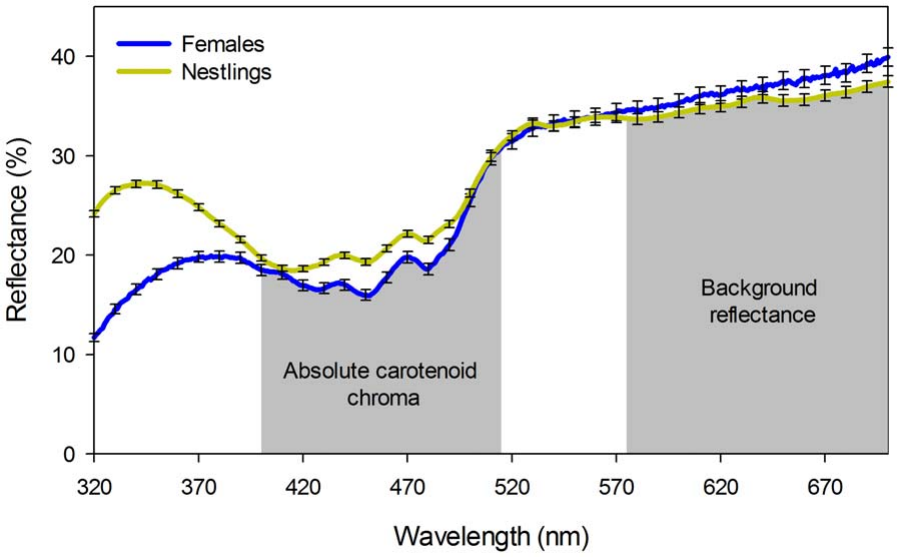
Reflectance curve of Great Tit yellow breast feathers measured by objective spectrophotometry. Mean value (± SE) for females (n = 40) and nestlings (n = 296) is given. The wavelengths used to calculate absolute carotenoid chroma (–1× (R_400−515_/R_575−700_)) and background reflectance (ΣR_575−700_) are highlighted in grey.

Absolute carotenoid chroma of nestlings averaged –0.570 (SD = 0.0681, n = 296) while in adult females it averaged –0.493 (SD = 0.0502, n = 40). Background reflectance of nestlings was on average 4459 (SD = 1196.8, n = 296) whereas that of adult females was on average 4651 (SD = 711.1, n = 40; Fig. 1).

### Pigment-based Component of Feather Colouration

**Figure 2:**
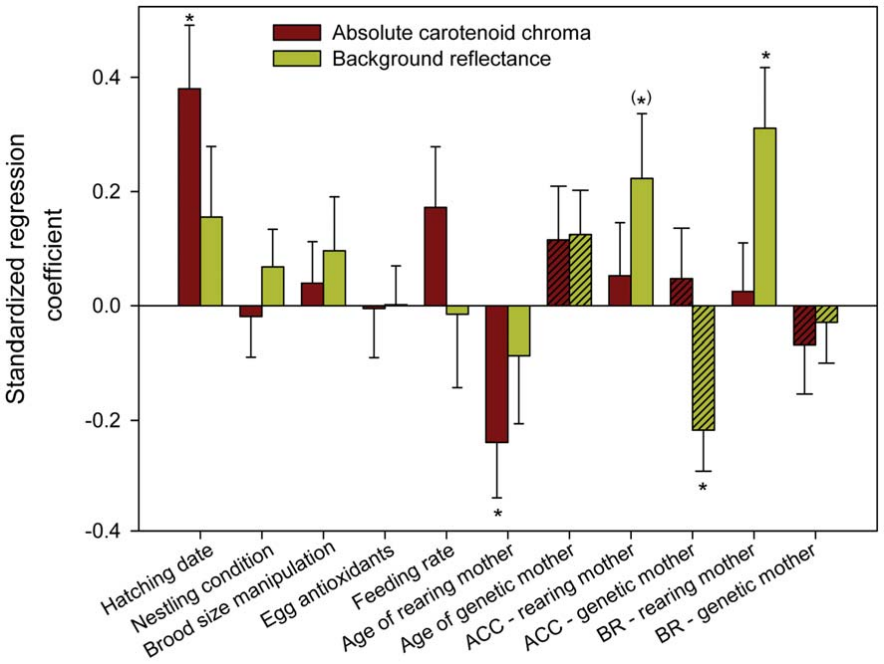
Standardized effects of genetic and environmental factors on yellow breast colouration of Great Tit nestlings. Depicted are regression coefficients (+ SE) of fixed factors from linear mixed models. Hatched bars depict effects of the genetic mother. Asterisks denote significance at *p<0.05, (*) p<0.06. ACC  =  absolute carotenoid chroma, BR  =  background reflectance. Parameter estimates are given for 1y old (relative to older) females and reduced (relative to enlarged) broods. Only fixed effect are included (a significant part of background reflectance is explained by nonspecific environmental conditions, represented by the random effect of the nest of rearing).

The absolute carotenoid chroma of nestlings increased during the season. Nestlings reared by older females also had higher absolute carotenoid chroma, indicating a higher concentration of carotenoids in feathers. Neither the pigment-based nor the structural component of the yellow breast feathers of rearing and genetic mothers predicted absolute carotenoid chroma of the nestlings. Similarly, neither the concentration of antioxidants in yolk nor the brood size manipulation affected absolute carotenoid chroma of nestlings. Only small amount of variation in absolute carotenoid chroma of nestlings was explained by the nest of rearing or the nest of origin (Table 1, Fig 2).

**Table 1:** Linear mixed model explaining absolute carotenoid chroma of yellow breast feathers in Great Tit nestlings.

Effect	Estimate	SE	Den. DF	F	P
FIXED EFFECT					
Intercept	−1.583	0.36	22.0		
Absolute chroma of rearing mother	0.071	0.13	25.3	0.3	0,58
Absolute chroma of genetic mother	0.066	0.12	29.4	0.3	0.59
Background reflectance of rearing mother	2.4×10^–6^	8.2×10^–6^	24.1	0.1	0.77
Background reflectance of genetic mother	–6.3×10^–6^	8.0×10^–6^	31.5	0.6	0.44
Age of rearing mother*	–0.032	0.01	25.5	6.0	**0.02**
Age of genetic mother*	0.016	0.01	32.3	1.5	0.23
Brood size manipulation**	0.005	0.01	17.6	0.3	0.59
Hatching date	0.009	2.5×10^–3^	21.9	11.7	**<0.01**
Yolk antioxidants, PC1***	–1.8×10^–4^	3.3×10^–3^	25.0	<0.1	0.96
Feeding rate per capita	0.007	4.6×10^–3^	31.4	2.6	0.12
Nestling condition	–0.001	4.7×10^–3^	223.0	0.1	0.79
Feather length	–0.007	2.4×10^–3^	245.0	8.0	**<0.01**
RANDOM EFFECT	Estimate	SE	% Var	Walds Z	P
Nest of rearing (Dyad)	2.4×10^–4^	2.8×10^–4^	5.3	0.9	0.20
Nest of origin (Dyad)	3.0×10^–4^	2.7×10^–4^	6.9	1.1	0.13
Dyad	2.7×10^–4^	3.7×10^–4^	6.2	0.7	0.23
Residual	0.004	3.3×10^–4^	81.4		

For fixed effects, type 3 tests and denominator DF are presented, numerator DF = 1 in all cases. For random effects, covariance parameter estimates are presented (REML method). Likelihood ratio test of the overall significance of random effects: χ^2^ = 15.09, DF = 3, P = 0.002. P-values of significant factors are in bold. Least squares means ± SE for nestling reared by 1y old females: −0.58±0.01, nestling reared by older females: −0.55±0.01.

* Estimate for 1y old (relative to older) females.

** Estimate for reduced (relative to enlarged) broods.

*** PC1 of yolk antioxidants included the concentrations of vitamin A, vitamin E, lutein and zeaxanthin in egg yolk; all concentrations were log_10_-transformed.

### Structural Component of Feather Colouration

Females with lower absolute carotenoid chroma and thus a lower carotenoid content of the yellow breast feathers produced nestlings with higher background reflectance. By contrast, a nearly significant positive relationship was observed between background reflectance of nestlings and absolute carotenoid colouration of the foster female. Background reflectance of nestlings increased with background reflectance of the rearing female, and was significantly explained by the nest of rearing. No other examined factor was significant (Table 2, Fig 2).

### Pigment-based vs. Structural Component

**Figure 3:**
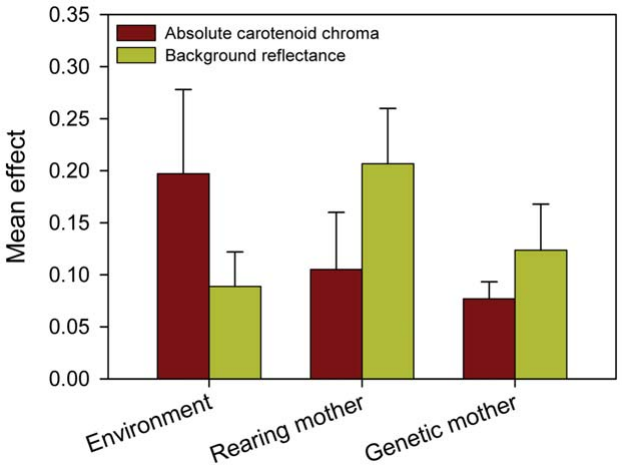
Overall effects of the rearing environment and rearing vs. genetic mother on colouration of Great Tit nestlings. Depicted are means (+ SE) of absolute values of standardized regression coefficients (see Fig. 2) to compare overall effect size regardless of effect direction. Environment includes hatching date, brood size manipulation and per-capita feeding rates. Effects of the rearing and the genetic mother include their age and colouration (absolute carotenoid chroma and background reflectance). Only fixed effect are included (a significant part of background reflectance is explained by nonspecific environmental condition, represented by the random effect of the nest of rearing).

Both pigment-based and structural components of nestling carotenoid colouration were related to the environmental conditions. However, the structural component of the feather colouration was also partially genetically determined. The pigment-based component seemed to depend on the rearing conditions related to food quality. By contrast, the structural component seemed to be related to the female colouration and to the general rearing environment which was not represented by the fixed factors included in our study (Tables 1, 2; Figs 2, 3).

## Discussion

In this study, we separated origin- vs. environment-related determinants of both the structural and the pigment-based components of carotenoid-based feather colouration. Great Tit nestlings reared by older mothers and those that hatched later in the season were more intensely yellow. Carotenoid chroma of nestlings was independent of the colouration of both the rearing and the genetic mother and of the antioxidant concentration in the egg yolk. On the other hand, the structural component of nestling colouration was related to the feather colouration of both the rearing and the genetic mother and to the nest of rearing, but not to the hatching date, the feeding rate or egg yolk antioxidants. Thus, the two components reflected different aspects of the nestling environment and only the structural component was related to the genetic/pre-natal factors. These results support the notion that the pigment-based and the structural components of feather colouration are determined independently [10], [15].

**Table 2:** Linear mixed model explaining background reflectance of yellow breast feathers in Great Tit nestlings.

Effect	Estimate	SE	Den. DF	F	P
FIXED EFFECTS					
Intercept	–6258.48	7031.07	23.5		
Absolute chroma of rearing mother	5306.85	2703.22	36.1	3.8	**0.06**
Absolute chroma of genetic mother	–5307.99	1757.94	23.1	9.1	**<0.01**
Background reflectance of rearing mother	0.52	0.18	31.5	8.6	**<0.01**
Background reflectance of genetic mother	–0.05	0.12	25.6	0.2	0.68
Age of rearing mother*	–208.08	284.71	33.3	0.5	0.47
Age of genetic mother*	298.35	184.96	26.6	2.6	0.12
Brood size manipulation**	230.24	226.02	19.8	1.0	0.32
Hatching date	62.26	49.56	23.4	1.6	0.22
Yolk antioxidants, PC1***	1.18	46.69	19.2	<0.1	0.98
Feeding rate per capita	–11.28	96.12	36.0	<0.1	0.91
Nestling condition	79.14	77.00	241.0	1.1	0.30
Feather length	3.29	38.62	261.0	<0.1	0.93
RANDOM EFFECTS	Estimate	SE	% Var	Walds Z	P
Nest of rearing (Dyad)	297099	153221	22.6	1.9	**0.03**
Nest of origin (Dyad)	11262	46395	0.9	0.2	0.40
Dyad	86195	140129	6.5	0.6	0.27
Residual	922179	84174	70.0		

For fixed effects, type 3 tests and denominator DF are presented, numerator DF = 1 in all cases. For random effects, covariance parameter estimates are presented (REML method). Likelihood ratio test of the overall significance of random effects: χ^2^ = 39.9, DF = 3, P<0.001. P-values of significant factor are in bold.

* Estimate for 1y old (relative to older) females.

** Estimate for reduced (relative to enlarged) broods.

*** PC1 of yolk antioxidants included concentrations of vitamin A, vitamin E, lutein and zeaxanthin in egg yolk; all concentrations were log_10_-transformed.

The expression of carotenoid-based signals is likely to be affected by a complex system of physiological trade-offs as well as non-physiological costs and benefits (e.g. predation risk or social interactions; see [60] for a review). However, the intensity of the chromatic component of the carotenoid-based colouration has been repeatedly shown to depend primarily on the carotenoid content of feathers. This in turn depends on the carotenoid access in the diet (see [7], [8] for a review). Here we demonstrated that carotenoid chroma of nestlings was related to two factors presumably connected with the quality of the food delivered by parents, namely the season and the age of rearing mother. Breast feathers of Great Tit nestlings hatched later in the season are often more yellow (e.g. [25], [27], [36]). This occurs despite the fact that the carotenoid content in their main food, caterpillars, tends to be stable or decrease in the season [61], [62]. However in our population, the concentration of egg yolk carotenoids increased with the season [56] suggesting that the carotenoid supply may have increased as well. Moreover, older foster mothers raised more chromatic nestlings as compared to 1y-old females, which could suggest that they were able to supply the young with higher-quality food. This is interesting in relation to a recent observation that Great Tits are able to discriminate food based on its carotenoid content [63]. Despite the experimental evidence that food access is critical for the expression of carotenoid-based colouration [64], but in accordance with a similar study in nestling Great Tits [28], we found no relationship between food quantity represented by feeding rate, and nestling breast colouration. Thus, although the prey biomass delivered to nestlings strongly increased with feeding rates in our population (V. Remeš, unpubl. data), it seems that the amount of food provided by parents was not limiting for the expression of nestling carotenoid colouration in our population. In sum, we were able to identify the specific environmental factors explaining variation in the chromatic component of the carotenoid-based signal, which were presumably related to the food quality. This result agrees with previous studies (see [7], [8] for a review).

Carotenoid-removal experiments revealed that the white structural background of carotenoid-coloured feathers is crucial for the production of yellow carotenoid-based displays [3], [10], [15]. It is produced by an incoherent scattering of all visible wavelengths from a nanostructure of keratin and air vacuoles. Structural colours have been suggested to have a limited condition-dependence [4]. However experimental studies are rare and their results are ambiguous (see [22] for details). Moreover, structural colours are produced by a diverse set of nanostructures, which are likely to differ in developmental mechanisms, so any generalization among different anatomical systems should be made with caution [4]. The reflectance properties of the structural white feather background remained unaffected by the experimentally induced fast moult in the Blue Tit [21] and the manipulation of food and carotenoid intake in the American Goldfinch *Carduelis tristis* [10]. By contrast, Great Tit nestlings raised in experimentally reduced broods developed the breast plumage with higher reflectance of the white background structure compared with nestlings from control broods [15]. Dark-eyed Juncos *Junco hyemalis* on a high-protein diet also grew brighter white tail patches than individuals on a low-protein diet [19]. Contradictory findings of experimental studies conducted up to now may reflect interspecific differences in condition-dependence, differences between the juvenal plumage ([15], this study), the first basic plumage [21], the first alternate plumage [10] and the feathers replacing removed feathers [19], or they may reflect sex-specific effects ([10], [19] studied only males, whereas [21], [15] studied both sexes). We found that the reflectance of the achromatic component of Great Tit nestlings was related to both the rearing environment and attributes of the genetic mother (her carotenoid chroma). Currently, we are not able to explain the mechanism of the antagonistic effect of the rearing vs. the genetic mother chromatic colouration on the nestling achromatic component. A substantial proportion of the variance in the structural component was explained by the random factor of the nest of rearing. Thus, in contrast with the pigment-based component, we were not able to identify specific factors explaining the structural component. This is especially puzzling in the case of brood size manipulation. Although it affected growth of the nestlings (see above), it had no effect on nestling colouration. It seems that structural component of nestling colouration is affected by environmental factors impacting other parameters of nestlings than body size (e.g. certain physiological systems). Overall, our results suggest that in our population the nestling feather colouration is under the control of both environmental and genetic/pre-natal effects.

Pigment-based and structural components of the carotenoid-based colouration are in our population of Great Tits subject to different levels of the environmental vs. the genetic/pre-natal determination. This may be mediated by a single coloured patch drawing from distinct biochemical pools, e.g. diet-derived carotenoids and synthesized keratins [5]. Hence, a single carotenoid patch may serve as a multicomponent signal, simultaneously conveying different aspects of the individual quality and history [65], [3], [10], [66], [52], [15], [24]. Display of multicomponent signals may provide a variety of benefits, including conveying more information to the receiver or an improvement of efficacy of transmission, reception, and processing of signals (see [67] for a review). However, it is important to stress that the two components can interact in a complex way and that multicomponent signals can be fully understood only by investigating their components simultaneously [68], [69]. For example, the brightness of feather structure interacts with its carotenoid content in colour production. High carotenoid concentration may produce intensive chroma only if the underlying feather structure is sufficiently reflective. Alternatively, if the structural component is very bright, this might make it necessary to add more carotenoids to the feather to get the same carotenoid chroma as in a feather with low structural reflectance. Thus, production of the most chromatic yellow signal might require a balance between these two components of colouration. These and similar questions remain virtually unexplored. However, the potential for the two components to interact in producing a visual signal is given by the perception system of birds, i.e., to what extent birds perceive the two components separately versus as one visual signal (e.g. [24]).

Juvenal coloration of Great Tit young became an important model system in the investigation of the expression of carotenoid colouration. However, it is important to note two caveats. First, it is not sure what the current function of the yellow breast coloration in Great Tit nestlings might be. Since breast feathers are moulted in autumn before subsequent breeding season [55], sexual selection cannot play a role. Juvenal breast coloration does not seem to affect parental favouritism when feeding the nestlings in the Great Tit ([70], [71], but see [72]). Similarly, there seems to be no natural selection on this trait after leaving the nest, at least in a Swiss population [73]. The possibility of correlated selection through adult plumage was also ruled out by the lack of correlation between juvenal and adult yellow breast coloration in the same population [28]. Second, extrapolation of results from the study of juvenal coloration to adult coloration might be troublesome. Studies of adult yellow coloration in titmice differ in the role they ascribe to genes versus environment in determining the expression of this trait (Great Tit [24], [38]; Blue Tit [43], [44]), which makes direct comparison with the studies conducted on nestlings difficult. Moreover, the function of yellow breast coloration in adult Great Tits is not clear either. Although some studies suggested that this colour patch might be a signal of individual quality [74], other studies found no functional significance [75], [76], and still others suggested that it might even have a function in crypsis [77].

To conclude, our study showed that the pigment-based and the structural components of the carotenoid-based colouration were determined differently. The chromatic component was related to specific environmental factors, whereas the achromatic component was related to both female-related and nonspecific environmental factors as well as genetic/pre-natal factors. Our results reinforce the hypothesis that the carotenoid-based colouration may serve as a multicomponent signal, with the chromatic and the achromatic components reflecting a different aspect of the individual’s quality and/or history. We suggest that further experimental studies focus on the effects of specific environmental and genetic factors on both the feather pigment content and the nanostructure in carotenoid-based feather patches. Future studies should also focus on the effects of the nanostructure and the pigment content on the reflectance of feathers [5]. Here, appropriate methods of scoring the feather reflectance/colouration need to be used, as different methods can generate different results (see [52]). The hypothesis that the variability in both components can be recognized by birds and that both components are used either separately or in interaction in communication needs to be verified by visual modelling and behavioural tests.

## Supporting Information

Figure S1 **The design of cross-fostering and brood size manipulation.** Each box represents a dyad consisting of two synchronously timed nests (points represent hypothetical nestlings). Nestlings within their nest of origin are ranked according to their weight from the heaviest to the lightest. We exchanged either even- or odd-ranked nestlings (exchange of even-ranked nestlings is depicted). The brood size was manipulated by two randomly chosen young: In one nest of the dyad one extra nestling was taken also to the foster nest. In the other nest of the dyad one extra nestling was left it in its nest of origin.
(TIF)
