# Supplementary figures and images for: Environmental and Genetic Effects on Pigment-Based vs. Structural Component of Yellow Feather Colouration
Source: PLoS One. 2012 May 10;7(5):e36640. doi: 10.1371/journal.pone.0036640 (PMC3349711; doi:10.1371/journal.pone.0036640)

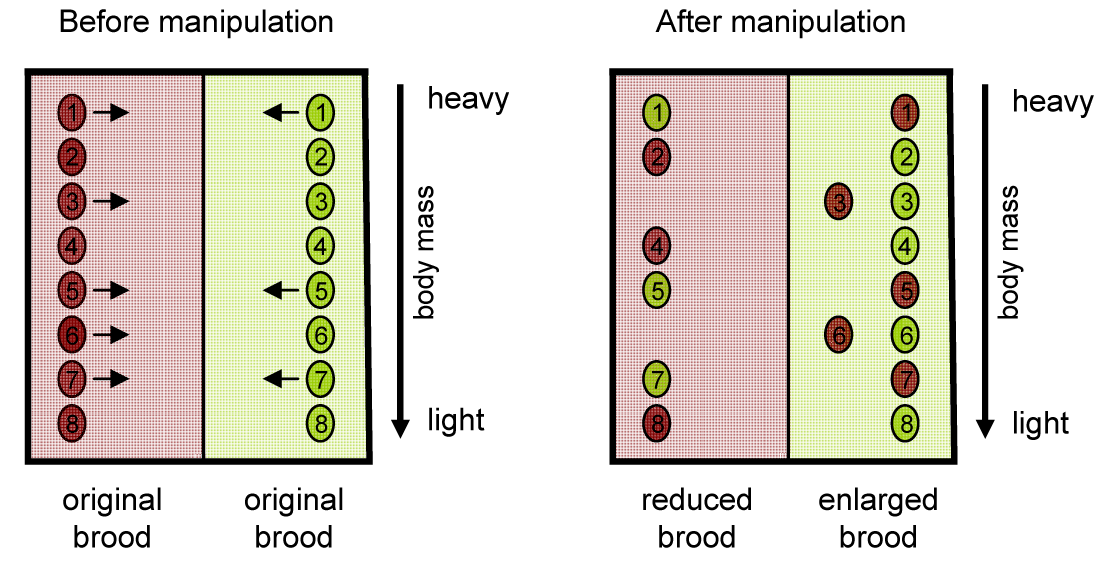

Supplement: Figure S1 — The design of cross-fostering and brood size manipulation. Each box represents a dyad consisting of two synchronously timed nests (points represent hypothetical nestlings). Nestlings within their nest of origin are ranked according to their weight from the heaviest to the lightest. We exchanged either even- or odd-ranked nestlings (exchange of even-ranked nestlings is depicted). The brood size was manipulated by two randomly chosen young: In one nest of the dyad one extra nestling was taken also to the foster nest. In the other nest of the dyad one extra nestling was left it in its nest of origin. (TIF) [file pone.0036640.s001.tif]
